# LRP1 facilitates Jamestown Canyon virus infection of neurons

**DOI:** 10.1128/jvi.01841-25

**Published:** 2025-11-28

**Authors:** Zachary D. Frey, David A. Price, Kaleigh A. Connors, Rachael E. Rush, Griffin Brown, Cade E. Sterling, Farheen Fatma, Safder S. Ganaie, Xiaoxia Cui, Zachary P. Wills, Gaya K. Amarasinghe, Daisy W. Leung, Amy L. Hartman

**Affiliations:** 1 Center for Vaccine Research, University of Pittsburgh School of Medicine 12317 , Pittsburgh, Pennsylvania, USA; 2 Department of Medicine, Washington University School of Medicine, St. Louis, Missouri, USA; 3 Department of Infectious Diseases and Microbiology, School of Public Health, University of Pittsburgh 6614, Pittsburgh, Pennsylvania, USA; 4 Department of Pathology and Immunology, Washington University School of Medicine, St. Louis, Missouri, USA; 5 Genome Engineering and Stem Cell Center (GEiC), Department of Genetics, Washington University School of Medicine, St. Louis, Missouri, USA; 6 Department of Neurobiology, University of Pittsburgh School of Medicine 12317 , Pittsburgh, Pennsylvania, USA; St Jude Children's Research Hospital, Memphis, Tennessee, USA

**Keywords:** Jamestown Canyon virus, primary rat neurons, LRP1, CD91, bunyavirus, host factor

## Abstract

Jamestown Canyon virus (JCV) is a bunyavirus and arbovirus that causes viral neuroinvasive disease in North America. JCV neuropathogenesis is understudied, and no pro-viral host factors for cellular infection have been identified. Here, we assessed the role of low-density lipoprotein receptor-related protein 1 (LRP1; also known as CD91), which has been identified as a host entry factor for other bunyaviruses, in mediating JCV infection with a focus on neurons. Both neuronal and non-neuronal immortalized cell lines deficient for murine Lrp1 displayed reduced binding, internalization, and infection with JCV. Furthermore, a soluble extracellular domain of human LRP1 can bind directly to JCV, and the same region of LRP1 can neutralize JCV infection. Primary neurons, where Lrp1 was highly expressed, were permissive for JCV infection. Treatment of primary neurons with the murine Lrp1 ligand receptor-associated protein (RAP) resulted in reduced infectivity with JCV. Finally, treatment of Lrp1 knockout cells with RAP further reduced JCV infection, suggesting that other low-density lipoprotein receptors may mediate JCV entry in the absence of Lrp1. Together, these results support LRP1 as an important cellular factor for efficient neuronal infection by JCV. Emerging support for the use of LRP1 for viral entry by multiple bunyaviruses also makes LRP1 a promising target for antiviral development.

**IMPORTANCE:**

Jamestown Canyon virus (JCV), an emerging mosquito-transmitted virus in North American white-tailed deer, causes several cases of severe neurologic disease in humans each year. Our results on the use of low-density lipoprotein receptor (LDLR)-related protein 1 by JCV for efficient cellular infection of neurons underscore the significance of the LDLR family of receptors in viral infection. Recent studies also highlight the emerging use of the LDLR family of receptors for virus entry by the bunyavirus and alphavirus family members. Defining cellular factors that mediate infection by mosquito-transmitted viruses is critically important to the prototype pathogen approach for combating infectious diseases and countermeasure development.

## INTRODUCTION

In North America, *Aedes*, *Culiseta*, and *Anopheles* mosquitoes transmit orthobunyaviruses (family *Peribunyaviridae*), including Jamestown Canyon virus (JCV), La Crosse encephalitis virus (LACV), and other California serogroup viruses (1–3). White-tailed deer are the likely reservoir host of JCV in the USA and Canada, and the high seroprevalence in both deer (~80%) and humans (up to 20%) in endemic regions highlights the zoonotic potential of this relatively understudied virus (4–9). JCV disease in humans is often asymptomatic or results in a mild febrile illness, but infection can progress to neuroinvasive disease with symptoms such as encephalitis and meningitis (10–13). In 2021, JCV was the third most prevalent locally acquired arbovirus in the USA, and 75% (24/32) of patients infected with JCV were hospitalized, leading to two deaths (14). Despite the potential for zoonotic spread and a high rate of hospitalization in reported human cases, there remains a major gap in understanding the mechanisms of cellular infection by JCV.

Host cell proteins within the low-density lipoprotein receptor (LDLR) family have garnered recent attention for their role in viral infections. One of the most structurally and functionally complex members of the LDLR family is low-density lipoprotein receptor-related protein 1 (human LRP1; murine Lrp1; also known as CD91). In foundational studies, we showed that LRP1 can act as a critical entry factor for Rift Valley fever virus (RVFV, *Phenuiviridae*) (15, 16). Subsequent studies with severe fever with thrombocytopenia syndrome virus (SFTSV, *Phenuiviridae*) and Oropouche orthobunyavirus (OROV, *Peribunyaviridae*) provide support for LRP1 as a pan-bunyavirus entry factor (17, 18). In parallel, recent work has shown that Crimean-Congo hemorrhagic fever virus (*Nairoviridae*) uses the related LDLR as an entry factor (19–21). Additional RNA viruses also rely on LRP1 for later stages of infection, including the orthobunyavirus LACV (22). Together, these studies highlight the greater implications for LDLR family members as broad-spectrum pro-viral factors.

LRP1 is a large ~600 kDa transmembrane protein that contains an extracellular alpha chain with four ligand-binding clusters (CL) separated by epidermal growth factor repeats and β-propeller domains, a transmembrane domain, and a cytoplasmic tail. The ligand-binding clusters are composed of cysteine-rich complement-type repeats (CR), with CL I–IV containing 2, 8, 10, and 11 CR repeats, respectively (23). Most ligands for LRP1 bind to CL II (CL_II_) and CL IV (CL_IV_), including the receptor-associated protein (RAP) (24). RAP is a high-affinity molecular chaperone for both LRP1 and other members of the LDLR family that prevents premature binding of ligands until the receptor localizes to the cell membrane (25). Domain 1 and domain 3 of RAP (RAP_D3_) can bind to LRP1, and RAP_D3_ is sufficient to perform the chaperone duties of the full-length protein by binding to both CL_II_ and CL_IV_ (26). Previous studies have shown that the surface glycoproteins of OROV and RVFV bind to CL_II_ and CL_IV_ of LRP1, with both viruses demonstrating an apparent greater preference for CL_IV_. Furthermore, OROV and RVFV likely have overlapping binding sites on LRP1, as a soluble form of RVFV glycoprotein Gn is able to competitively inhibit OROV infection *in vitro* (15, 18).

LRP1 is highly expressed in multiple brain cells, including neurons, astrocytes, and microglia, and is implicated in normal neurodevelopment and multiple neurodegenerative diseases (27–31). The role of LRP1 in mediating neuronal infection by neurotropic bunyaviruses, such as JCV, remains unexplored. In this study, we explore the breadth of LRP1 usage by bunyaviruses and provide insights into the mechanism by which *Peribunyaviridae* use LRP1 for infection of neurons, a primary target of infection. We found that LRP1 is needed for early-stage entry of JCV into different cell types, and that blocking or neutralizing LRP1 binding sites reduces JCV infection of primary neurons. These findings highlight the role that LRP1 plays in JCV infection of neurons and further underscore LRP1 as a multi-bunyavirus host factor.

## MATERIALS AND METHODS

### Biosafety

All work with JCV, OROV, and ZIKV was performed at biosafety level 2 in accordance with the University of Pittsburgh biosafety guidelines. All work with RVFV ZH501 was performed at biosafety level 3+ in the Regional Biocontainment Laboratory (RBL) at the University of Pittsburgh by approved personnel. The University of Pittsburgh RBL is registered with the Centers for Disease Control and Prevention and the United States Department of Agriculture for work with RVFV.

### Viruses

The following viruses were obtained through the NIH Biodefense and Emerging Infections (BEI) Research Resources Repository, NIAID, NIH: Jamestown Canyon virus (strain 61V-2235; NR-536), Zika virus (strains PRVABC59; NR-50684 and MR766; NR-50065). Oropouche virus (strain BeAn19991) was rescued from reverse genetics as previously described (32) and was kindly provided by Paul Duprex and Natasha Tilston-Lunel (University of Pittsburgh Center for Vaccine Research). The ZH501 strain of RVFV was kindly provided by Barry Miller (CDC, Fort Collins, Colorado) and Stuart Nichol (CDC, Atlanta, Georgia) and was rescued through reverse genetics as previously described (33). Viruses were propagated in Vero E6 cells with standard culture conditions using standard D2 media comprised of Dulbecco’s modified Eagle’s medium (DMEM) supplemented with 1% penicillin/streptomycin (Pen/Strep), 1% L-glutamine (L-Glut), and 2% fetal bovine serum (FBS). A standard viral plaque assay (VPA) was used to determine the infectious titer of the stocks. The agar overlay for the VPA was comprised of 1× minimal essential medium, 2% FBS, 1% Pen/Strep, 1% HEPES buffer, and 0.8% SeaKem agarose (Fisher, BMA5000); the assay was incubated at 37°C for 3 (JCV, OROV, RVFV) or 5 (ZIKV) days, followed by visualization of plaques with 0.1% crystal violet. Viral passage 1 or 2 was used for the enclosed experiments.

### Cell lines

Vero E6 (ATCC, CRL-1586) and BV2 cells were cultured in DMEM (ATCC, 30-2002) and supplemented with 1% Pen/Strep, 1% L-Glut, and 10% FBS. N2a cells were maintained in Eagle’s Minimum Essential Medium (ATCC, 30-2003) supplemented with 1% Pen/Strep, 1% L-Glut, and 10% FBS. BV2 and N2a LRP1 knockout (KO) cell lines were generated and validated as previously described (15) and maintained in the same culture media as their wild-type (WT) counterparts.

### Lrp1-deficient cell line infections

N2a and BV2 cell lines deficient for murine Lrp1 were previously described and validated (15). KO cells and their wild-type counterparts were seeded into 24-well plates at 1–2 × 10^5^ cells/well. On the day of infection, media were removed from each well and replaced with 100 µL of virus diluted to an MOI of 0.1 in standard D2 media. The virus was incubated at 37°C for an hour, rocking every 15 min to ensure the monolayer did not dry out. Following the 1 h adsorption period, the inoculum was removed, and the cells were washed once with 1× PBS. Fresh D2 media were added, and the cells were incubated for 24 h prior to supernatant collection for measurement of viral RNA (vRNA) or infectious titers.

### Binding and internalization assays

Lrp1 KO or WT cells were seeded in 24-well plates at 1 × 10^5^ cells/well 1 day prior to infection. On the day of infection, media were removed and replaced with 200 µL of 10 µM surfen (34) or vehicle control (DMSO) in PBS. Cells were incubated for 30 min at 4°C. Following the incubation, surfen solution was removed and replaced with 200 µL of virus diluted to an MOI of 0.1 in standard D2 media. Plates were returned to 4°C for an hour. The inoculum was removed, and cells were washed five times with PBS containing 3% bovine serum albumin (BSA, Sigma, A3294) and 0.02% Tween-20. Binding samples were collected by adding 1 mL of Trizol (Fisher, 15-596-018) directly to the cell monolayer. For internalization assays, wells not collected for binding were incubated for 1 h in fresh D2 media at 37°C. Cells were washed once with the same wash buffer containing BSA + Tween-20, and samples were collected by adding 1 mL of Trizol directly to the cell monolayer.

### Immunofluorescence

Coverslips were fixed and virus inactivated in 4% paraformaldehyde for 15 min prior to storage in 1× PBS at 4°C prior to staining. Cells were permeabilized with 0.1% Triton X-100 diluted in 1× PBS for 10 min at room temperature. After permeabilization, coverslips were blocked in 5% normal goat serum (Thermo Fisher, 50062Z) for an hour at room temperature. Coverslips were incubated for 1 to 2 h at room temperature with primary antibodies. Samples were then incubated for an hour with secondary antibodies conjugated to a fluorophore. Coverslips were counterstained with Hoechst 33258 (Invitrogen, #H1398, 1:1000) and mounted on slides using Gelvatol (provided by the Center for Biologic Imaging). Fluorescent slides were imaged on either a Nikon A1 confocal microscope at the Center for Biologic Imaging, or a Leica DMI8 inverted fluorescent microscope at the Center for Vaccine Research. Images were processed using Fiji (v.1.53). The following antibodies were used for immunofluorescent staining during this study: mouse anti-βIII-tubulin (1:500; R&D Systems, MAB1195), custom rabbit anti-JCV-nucleoprotein (N) (1:500; Genscript), rabbit anti-LRP1 (1:500; Abcam, ab92544), antisera from mice immunized with a sublethal dose of JCV (1:200; generated in house), custom rabbit anti-OROV N (1:500; Genscript), mouse anti-ZIKV NS1 (1:500; Invitrogen, MA5-24585), goat anti-rabbit 488 (1:500; Invitrogen, A11008), goat anti-mouse 488 (1:500; Invitrogen, A11001), goat anti-rabbit 594 (1:500; Invitrogen, A11012), and goat anti-mouse 594 (1:500; Invitrogen, A11005).

### Quantification of viral RNA

RNA isolation was performed using an Invitrogen PureLink RNA/DNA kit (Fisher, 12-183-025) with a modified protocol as previously described (35). Briefly, supernatant was lysed in Trizol (Invitrogen, 15596026) at a dilution of 1:10 (100 µL sample, 900 µL Trizol). Then, 200 µL of chloroform was added to each sample, mixed, and then centrifuged at 12,000 × *g* for 15 min at 4°C to separate the aqueous and organic phases. The aqueous phase was removed and added to an equivalent volume of 70% ethanol. The PureLink RNA kit protocol was then followed for the remainder of the isolation, and RNA was eluted in 40 µL of RNase-free water. RT-qPCR was performed using the SuperScript III Platinum One-Step RT-qPCR Kit (Thermo Fisher, 11745-500), following a previously described protocol (35).

Primers targeting the JCV L-segment include 5′-CCTAGATGCTCCGTTGTCTATG-3′ (Jamestown-2364For) and 5′-TGCATTATTGGTGTGTGTTTGT-3′ (Jamestown-2448Rev). The TaqMan probe used includes (Jamestown-2387 Probe 5′ 6-FAM/TCAGTACAGTGGGATTAGAAGCTGGGA/BHQ_1 3′).

Primers targeting ZIKV PRVABC59 NS2B region include 5′-CTGTGGCATGAACCCAATAG-3′ (ZIKVPRABC59-4513For) and 5′-ATCCCATAGAGCACCACTCC-3′ (ZIKVPRABC59-4603Rev). The TaqMan probe includes (ZIKVPRABC59-4558Probe 5′ 6-FAM/CCTTTGCAGCTGGAGCGTGG /BHQ_1 3′).

Primers targeting the OROV S-segment include 5′-TACCCAGATGCGATCACCAA-3′ (OROV19991_For) and 5′-TTGCGTCACCATCATTCCAA-3′ (OROV19991_Rev). The TaqMan probe includes (OROV19991_Probe 5′ 6-FAM/TGCCTTTGGCTGAGGTAAAGGGCTG /BHQ_1 3′).

Primers targeting the RVFV L-segment include 5′-TGAAAATTCCTGAGACACATGG-3′ (RVFV-2912Fwd) and 5′-ACTTCCTTGCATCATCTGATG-3′ (RVFV-2981Rev). The TaqMan probe includes (RVFV-2950-Probe 5′ 6-FAM/CAATGTAAGGGGCCTGTGTGGACTTGTG /BHQ_1 3′).

### Neutralization with LRP1-Fc proteins

Vero E6 cells were seeded into 96-well plates at 2 × 10^4^ cells/well and allowed to incubate overnight. The day of infection, human LRP1 Cluster II-Fc (R&D Systems, 2368-L2), LRP1 Cluster IV-Fc (R&D Systems, 5395-L4B), and human IgG Fc control (R&D Systems, 110-HG) were diluted to 20 µg/mL in DMEM containing 1% Pen/Strep and 1% L-Glut and serially diluted 1:2. An equivalent volume of media containing 100–200 FFU of virus was added, and the virus mixture was incubated at 37°C for 1 h. Following the incubation, the media were removed from the cells, and 100 µL of the virus-protein mixture was added to the cells for 1 h at 37°C. Virus inoculum was removed, and an overlay comprised of 1.5% carboxymethylcellulose (CMC, Sigma, C4888), 0.5% Pen/Strep, and 5% FBS was added to the cells. The assay was incubated at 37°C for 18 h (JCV and OROV) or 42 h (ZIKV). CMC was removed, and cells were fixed for 15 min at room temperature with 4% PFA. Cells were stained according to the above immunofluorescence protocol with an antibody against viral nucleoprotein (JCV and OROV) or NS1 (ZIKV) and scanned using the Biotek Cytation 5 (Agilent). Foci were quantified using ImageJ.

### Biolayer interferometry (BLI) experiments

Vero E6 cells were infected with JCV, and the supernatant was harvested by centrifugation 2 days post-infection. The titer was estimated to be 1 × 10^6^ PFU/mL. One milliliter of the supernatant was placed in a 24-well plate and subjected to UV irradiation for 45 min for virus inactivation. Virus inactivation was previously validated through evaluation of post-inactivated virus growth under standard culture conditions. The supernatant was concentrated (12,000 × *g*, 10 min) to a final volume of 25 µL prior to dilution with PBS (pH = 7.4) supplemented with 1 mg/mL BSA and 0.05% Tween-20.

BLI assays were conducted at 30°C at 1,000 rpm (Octet Red, ForteBio). Anti-Human IgG Fc Capture biosensors were hydrated in PBS (pH = 7.4) supplemented with 1 mg/mL BSA and 0.05% Tween-20 for 15 min. Recombinant human LRP1 CL_IV_-FcHis, recombinant human LRP1 CL_II_-FcHis, or recombinant human IgG1 Fc (R&D Systems, #110-HG-100) were loaded at 100 nM for 500 seconds prior to baseline equilibration for 300 seconds. Association and dissociation of JCV were measured for 600 seconds. Data were baseline subtracted using sensors in buffer alone. Experiments were done in triplicate.

### Animal work

Timed-pregnant Long Evans (Crl:LE) rats were purchased from Charles River Laboratories (Wilmington, MA, USA). Fetuses obtained from embryonic day 18 dams were euthanized to obtain the neurons used in this study.

### Primary neuron culture

On the day prior to neuron isolation, acid-washed coverslips were coated with PDL/Laminin (Sigma, P7405-5MG; Invitrogen, 23017-015). Dissociation media (DM), comprised of Hanks’ Balanced Salt Solution (Invitrogen, 14175-103) supplemented with 10 mM anhydrous MgCl_2_ (Sigma, M8266), 10 mM HEPES (Sigma, H3375), and 1 mM kynurenic acid, was prepared. DM was brought to a pH of 7.2 and sterile filtered prior to use. On the day of isolation, a trypsin solution containing a few crystals of cysteine (Sigma, C7352), 10 mL of DM, 4 µL 1N NaOH, and 200 units of papain (Worthington, LS003126) and a trypsin inhibitor solution containing 25 mL DM, 0.25 g trypsin inhibitor (Fisher, NC9931428), and 10 µL 1N NaOH were prepared and filter sterilized. At embryonic day 18, dams were euthanized via CO_2_ inhalation overdose (primary method) followed by cervical dislocation (secondary method), and the brains of the embryos were removed and dissected. The cortices were separated from the hippocampus and placed into DM. Five milliliters of trypsin solution was added, and cortices were placed in a 37°C water bath for 4 min, swirling occasionally to mix. The trypsin solution was removed, and cortices were immediately washed with trypsin inhibitor once, and then twice more while swirling in the water bath. Following the washes, the trypsin inhibitor was removed and replaced with 5 mL of Neurobasal/B27 media, then triturated to dissociate the neurons. The final volume was brought to 10 mL of Neurobasal/B27, and cells were counted and plated at a density of 1.5 × 10^5^ neurons/well for 24-well plates, or 2.5–3 × 10^5^ neurons/well for 12-well plates. One hour after isolation, the media were removed and replaced with fresh Neurobasal/B27 media. Primary neuron cultures were maintained in Neurobasal/B27 media, which consists of standard Neurobasal Plus Medium (Thermo Fisher, A3582901) supplemented with 1% Pen/Strep, 1% L-Glut, and 2% B27 Plus Supplement.

### Viral growth curve infection

Primary rat neurons were maintained in culture for 3 days following isolation. Infection occurred on day 4 *in vitro*. JCV or ZIKV was thawed and diluted in D2 media to the desired MOI. Media were removed from wells, and 100 µL of inoculum was added to each well. Cells were incubated at 37°C for an hour, rocking every 15 min to prevent the monolayer from drying out. Following the adsorption period, the inoculum was removed from the wells and replaced with Neurobasal/B27 media. Cells were incubated for 15 min, and 100 µL of supernatant was inactivated in 900 µL of Trizol Reagent (Invitrogen, 15596026) to measure 0 h post-infection (hpi) viral RNA levels. At the appropriate time points, 100 µL of supernatant was inactivated in 900 µL of Trizol, the remaining supernatant was collected and stored at −80°C, and plates were fixed with 4% PFA for 15 min and stored at 4°C in 1× PBS for immunofluorescent staining.

### Western blot

Cells were inactivated in 100 µL of radioimmunoprecipitation assay buffer (Thermo Fisher Scientific, 89901) with 1% Halt Protease Inhibitor (Thermo Fisher Scientific, 78429) for 10 min at room temperature. Samples were centrifuged at 13,500 relative centrifugal force for 20 min. Cellular debris was removed, and a bicinchoninic acid (BCA) assay was completed following the manufacturer’s instructions (Thermo Fisher Scientific, Pierce BCA Protein Assay, 23227). Five micrograms of protein from each sample was loaded into a NuPAGE 4 to 12% Bis-Tris gel (Invitrogen, NP0323BOX) and run for 35 min at 165 V. The protein was transferred to a nitrocellulose membrane (LI-COR, 926-31090) using an iBlot 2 system (Invitrogen, IB21001). Membranes were blocked for 1 h, rocking at room temperature, in Intercept (PBS) Blocking Buffer (LI-COR, 927-70001). Following the block, membranes were incubated overnight rocking at 4°C with primary antibodies diluted in Intercept T20 (PBS) Antibody Diluent (LI-COR, 927-75001). The following primary antibodies were used in this study: mouse anti-GAPDH (1:1,000; Invitrogen, MA1-16757), rabbit anti-LRP1 (1:500; Cell Signaling, 64099S), custom rabbit anti-JCV-N (1:500; Genscript, Y743THG190-16), mouse anti-βIII-tubulin (1:500; R&D Systems, MAB1195), anti-RVFV Gn Clone 4D4 (1:500; BEI Resources, NR-43190), and mouse anti-β-actin (1:500; Santa Cruz Biotechnology, sc-47778). The following day, the membranes were washed by rocking in 10 mL of PBS-T three times for 5 min each. Membranes were probed for 1 h, rocking at room temperature, with either goat anti-rabbit IRDye 800CW (1:10,000; LI-COR, 926-32211), goat anti-rabbit IRDye 680RD (1:10,000; LI-COR, 925-68071), goat anti-mouse IRDye 800CW (1:10,000; LI-COR, 925-32210), or goat anti-mouse IRDye 680RD (1:10,000; LI-COR, 926-68070) diluted in Intercept T20 (PBS) Antibody Diluent (LI-COR, 927-75001). The membranes were washed by rocking in 10 mL of PBS-T three times for 5 min each, then rinsed with 1× PBS. The membrane was visualized using an Odyssey CLx Imager (LiCor, Lincoln, Nebraska, USA).

### Viral plaque assay

Vero E6 cells were plated into 12-well plates and allowed to incubate overnight until near confluency. Samples were serially diluted in D2 media. The inoculum was allowed to incubate for 1 h at 37°C and then removed. Agar overlay composed of 1× minimal essential medium, 2% FBS, 1% Pen/Strep, 1% HEPES buffer, and 0.8% SeaKem agarose (BMA5000) was added to each well. The assay was incubated at 37°C for 3 (JCV) or 5 (ZIKV) days to allow for the formation of plaques, fixed with 37% formaldehyde for at least 3 h, and then stained with 0.1% crystal violet for visualization and counting of plaques.

### Recombinant protein expression and purification

Murine mRAP_D3_ or mRAP_D3_ (K265A/K279E) expression plasmids were transformed into BL21(DE3) *E. coli* cells (Novagen). Colonies were cultured in Luria Broth media at 37°C to an OD_600_ of 0.6 and induced with 0.5 mM isopropyl-β-D-thiogalactoside for 14 h at 18°C. Cells were harvested and resuspended in lysis buffer containing 25 mM sodium phosphate (pH 7.5), 500 mM NaCl, 20 mM imidazole, 5 mM 2-mercaptoethanol, and were lysed using an EmulsiFlex-C5 homogenizer (Avestin). Lysates were clarified by centrifugation at 24,000 × *g* at 4°C for 40 min. Proteins were purified using a series of chromatographic columns as described previously (15). Protein purity was determined by Coomassie staining of SDS-PAGE. Soluble RVFV Gn was expressed in the same manner as mRAP_D3_ and resuspended in a lysis buffer containing 20 mM Tris-HCl (pH 8.0), 500 mM NaCl, and 5 mM 2-mercaptoethanol. Following lysis, the Gn pellet was resuspended in 20 mM Tris-HCl (pH 8.0), 500 mM NaCl, 5 mM imidazole, 8 M urea, and 1 mM 2-mercaptoethanol. RVFV Gn was refolded on a NiFF (GE Healthcare) column using a reverse linear urea gradient and eluted with imidazole. Gn was further purified using a size-exclusion column (SD200 10/300L, GE Healthcare).

### Competitive inhibition assays with mRAP_D3_ or RVFV Gn

Primary rat neurons were isolated as described above and maintained in culture for 3 days. Treatment and infection occurred on day 4 *in vitro*. Proteins were diluted to the desired concentration in both D2 and Neurobasal media. Culture media were partially removed and replaced with Neurobasal containing mRAP_D3_ or RVFV Gn. Plates were allowed to incubate for 45 min at 37°C. Following pre-treatment, all culture media were removed and replaced with D2 containing viral inoculum and either mRAP_D3_ or RVFV Gn. Plates were incubated for an hour, with rocking every 15 min. The inoculum was removed following the adsorption period, and Neurobasal containing mRAP_D3_ or RVFV Gn was added to the wells, and cells were returned to the incubator. Twenty-four hours (JCV) or 48 h (ZIKV) later, supernatant was collected, and plates were fixed with 4% PFA for 15 min, or cells were lysed with RIPA buffer for 10 min. Viral titers were then analyzed by RT-qPCR or VPA, and viral antigen was visualized through immunofluorescence staining or Western blot.

### Statistics and data analysis

Statistical analysis was performed using GraphPad Prism version 8.0. Significance was determined by one-way or two-way ANOVA. Error bars show mean and standard deviation. Significance is indicated by *, *P* < 0.05; **, *P* < 0.01; ***, *P* < 0.001; ****, *P* < 0.0001; ns, not significant.

## RESULTS

### Reduced infectivity of JCV in cells lacking Lrp1

**Fig 1:**
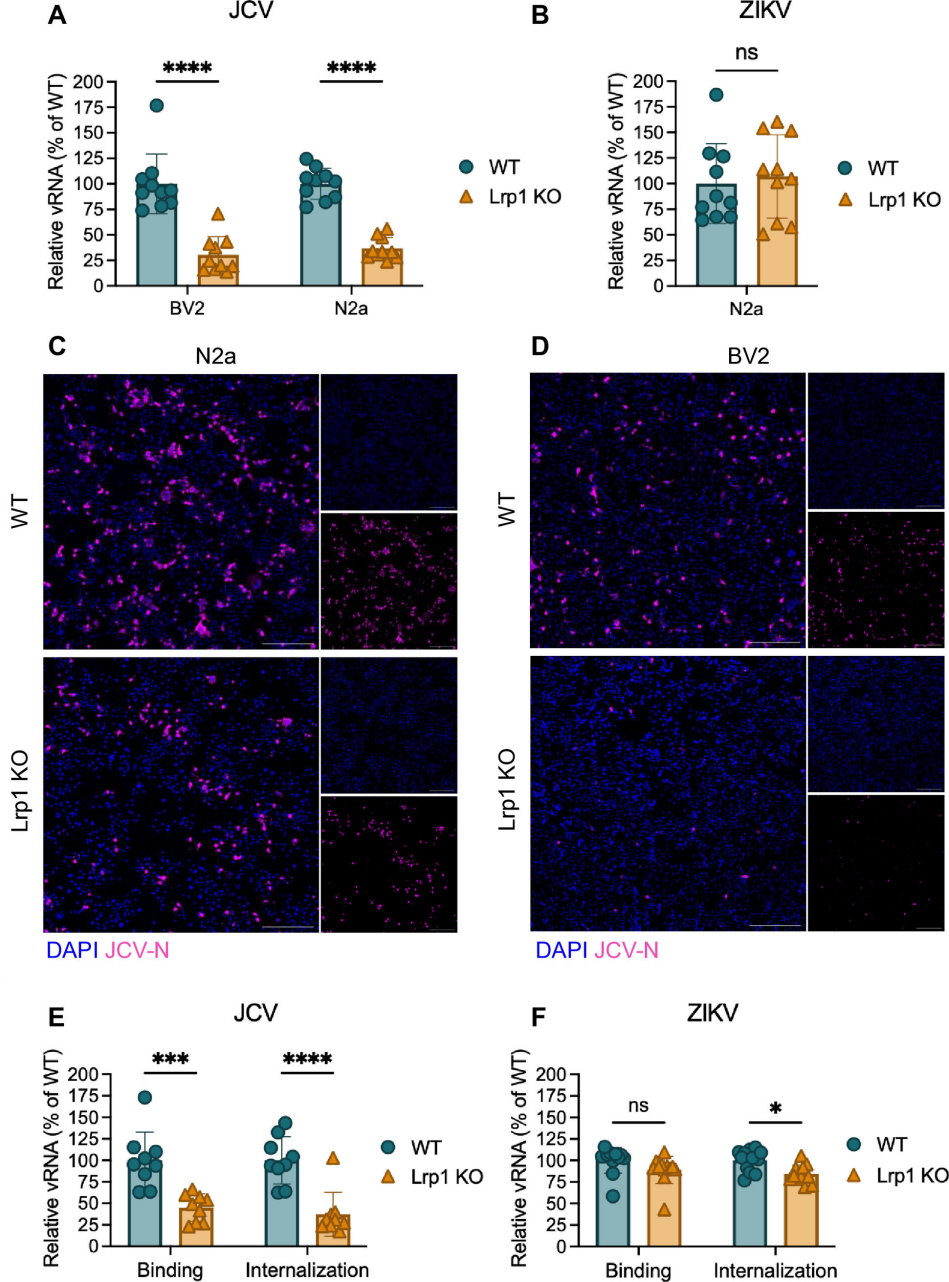
Lack of Lrp1 reduces JCV binding, internalization, and infection of murine cells. (**A, B**) Virus infectivity was determined by infecting WT and Lrp1 KO cells at an MOI of 0.1 with either (**A**) JCV or (**B**) ZIKV and quantifying viral RNA in the supernatant at 24 hpi (JCV) or 48 hpi (ZIKV). (**C, D**) JCV-N antigen (pink) counterstained with DAPI (blue). Slides were imaged at 10× using a Leica DMI8 inverted microscope. Scale bar = 250 µm. (**E, F**) Binding and internalization assays after surfen treatment using (**E**) JCV and (**F**) ZIKV as a control. Statistics were determined by two-way ANOVA with Dunnett’s multiple comparisons test (**A, E, F**) or Welch’s *t*-test (**B**). *, *P* < 0.05; ***, *P* < 0.001; ****, *P* < 0.0001; ns, no significance.

Using murine BV2 (microglia) and N2a (neuroblastoma) clonal cell lines with Lrp1 deletion (KO) (15, 18), the Lrp1-deficient cells and their Lrp1-sufficient counterparts were infected with JCV (MOI = 0.1), and the amount of viral RNA in the supernatant at 24 hpi was measured by RT-qPCR (Fig. 1A). There was a significant reduction in viral RNA production in the absence of Lrp1 in both cell types. We observed no reduction in infection when the KO N2a cells were infected with Zika virus (ZIKV; PRVABC59) as a control (Fig. 1B). BV2 cells do not support productive ZIKV infection and thus were not tested (18). The reduction in infection by JCV was visible by immunofluorescence microscopy, where both N2a and BV2 Lrp1 KO cells displayed decreased staining for JCV-N at 24 hpi compared to the WT cells (Fig. 1C and D).

### Reduced binding and internalization of JCV in Lrp1 KO cells

Because LRP1 functions as an entry factor early in the infection process for both RVFV and OROV, we performed binding and internalization assays using BV2 cells to investigate if LRP1 is involved in the early stages of JCV infection. BV2 WT or BV2 Lrp1 KO cells were first treated with the glycosaminoglycan antagonist surfen to prevent any non-specific binding to proteoglycans (34), incubated with JCV (MOI = 0.1) for 1 h at 4°C to allow binding but not internalization, and washed extensively before collection and RNA quantification by RT-qPCR. For internalization assays, cells were incubated at 37°C for another 1 h after washing. We observed a 50%–60% reduction in both binding and internalization in BV2 Lrp1 KO cells when compared to the WT cells (Fig. 1E). In contrast, we observed no reduction in binding and only a slight decrease in internalization of ZIKV in the KO cells (Fig. 1F). Reductions in binding and internalization were still evident in control experiments repeated without surfen, albeit to a lesser degree (Fig. S1).

### LRP1 CL_IV_ receptor decoy binds and neutralizes JCV

**Fig 2:**
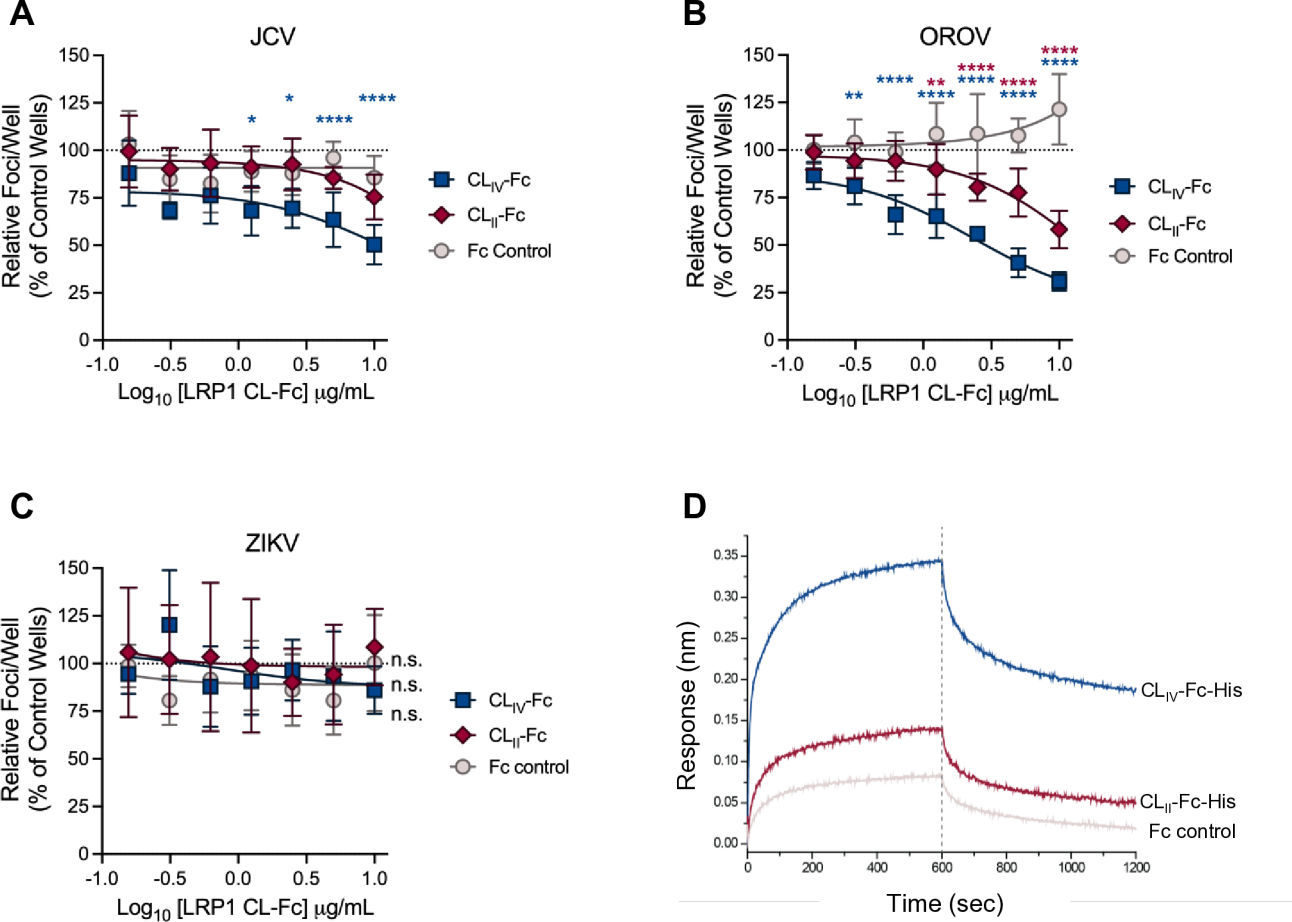
CL_IV_ of human LRP1 binds and neutralizes JCV. (**A–C**) FRNT assays were performed by mixing virus with increasing concentrations of LRP1 CL_II_-Fc, LRP1 CL_IV_-Fc, or an Fc control protein prior to infection of Vero E6 cells. Twenty-four hours later, foci were visualized by immunostaining, imaged using a Cytation 5, and quantified using ImageJ. Statistics were determined by two-way ANOVA with Dunnett’s multiple comparisons test. (**D**) Biolayer interferometry sensograms displaying association and dissociation of JCV virions to immobilized LRP1 CL_IV_-Fc-His (blue), CL_II_-Fc-His (red), or recombinant human IgG1 Fc (gray). Data were baseline subtracted using sensors in buffer alone. BLI experiments were performed in triplicate. *, *P* < 0.05; **, *P* < 0.01; ****, *P* < 0.0001; ns, no significance.

Our data point to a role for LRP1 in the entry stage of JCV infection; therefore, we assessed the ability of soluble fragments of the human LRP1 ectodomain (“receptor decoys”) to neutralize JCV infection in a focus reduction neutralization test (FRNT). Due to the large size of the entire LRP1 ectodomain, we used individual cluster domains fused to human IgG1 Fc, whereby exogenous addition of these ectodomain proteins was able to neutralize RVFV and OROV infection (15, 18). We pre-incubated human LRP1 CL_II_-Fc, CL_IV_-Fc, or an Fc control with 100–200 FFU of each indicated virus for 1 h prior to infection of Vero E6 cells. We used OROV and ZIKV as positive and negative controls for the assay, respectively. At 18 hpi (JCV and OROV) or 42 hpi (ZIKV), cells were fixed and stained for viral antigen to enumerate foci and compared the number of foci to control (untreated) wells. LRP1 CL_IV_-Fc neutralized JCV, but CL_II_ displayed little to no neutralization at the highest dose tested, suggesting better binding and/or a higher affinity of JCV for CL_IV_ of LRP1. We observed no reduction in JCV infection after treatment with the Fc control protein (Fig. 2A). OROV, our positive control in this assay, was neutralized by both CL_II_ and CL_IV_, confirming our previous findings (Fig. 2B) (18). As a negative control, we observed no reduction in infection when the assay was repeated with ZIKV (Fig. 2C). Furthermore, and consistent with our neutralization data, JCV showed increased binding to LRP1 CL_IV_-Fc compared to LRP1 CL_II_-Fc or an IgG-Fc control using biolayer interferometry (Fig. 2D). These findings suggest that soluble LRP1 CL_IV_ can directly neutralize JCV and reduce entry into cells, and that JCV binds directly to this cluster of LRP1.

### Primary neurons are permissive to JCV infection and express Lrp1

**Fig 3:**
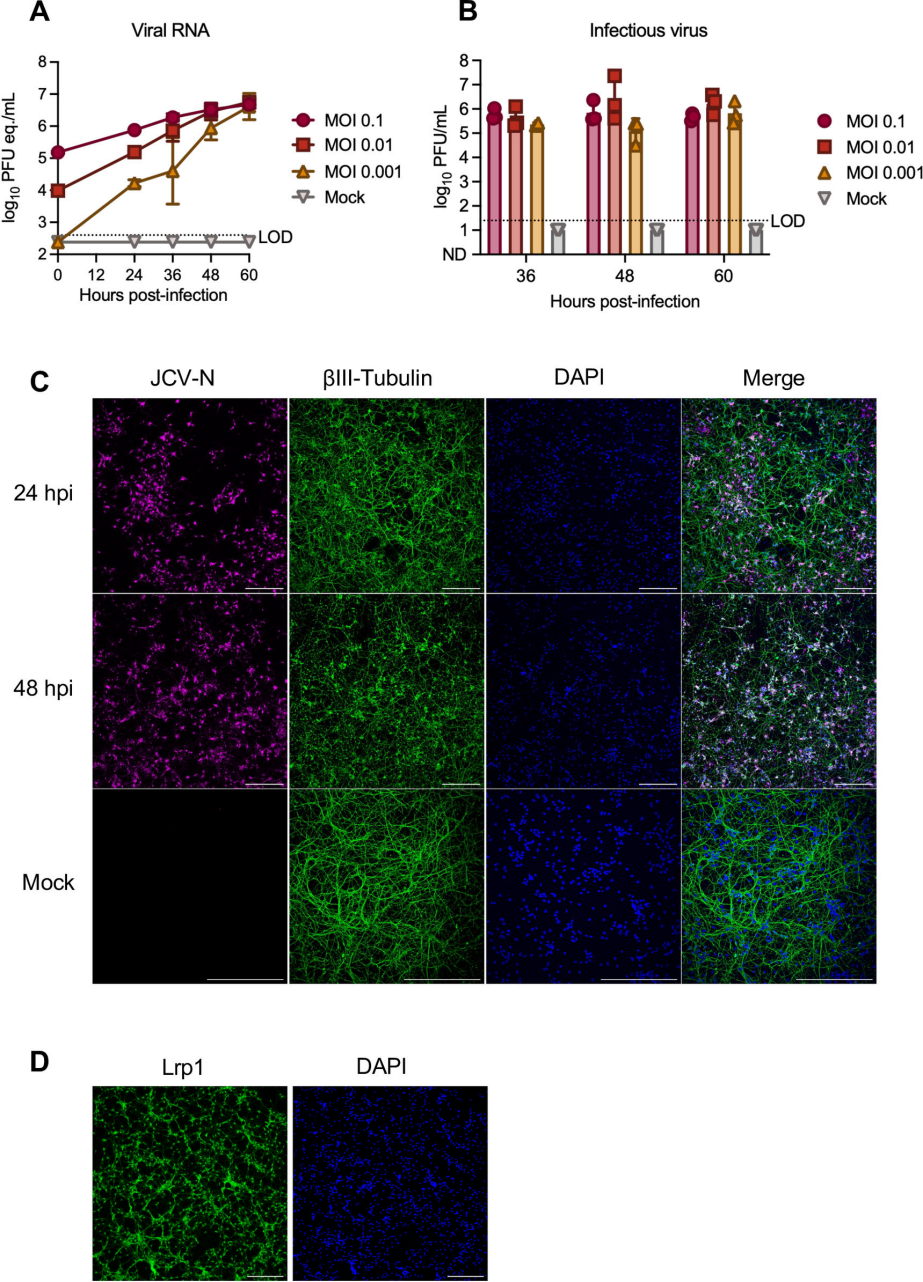
Replication kinetics of JCV in primary rat neurons. Primary rat neurons were infected with JCV at the indicated MOI. (**A**) Viral RNA or (**B**) infectious virus was quantified at 24, 36, 48, and 60 hpi time points. (**C**) Infected or mock-infected coverslips were stained for JCV-N (pink) and βIII-tubulin (green) and counterstained with DAPI (blue). Slides were imaged at 20× using a Nikon A1 confocal microscope. Scale bar = 250 µm. (**D**) Immunofluorescent microscopy of neurons after 4 days of culture. Coverslips were stained for Lrp1 (green) and counterstained with DAPI (blue). Slides were imaged at 10× using a Leica DMI8 inverted microscope. Scale bar = 250 µm.

Among humans who develop reportable clinical disease due to JCV infection, neurological issues are a primary manifestation. Despite this, few experimental studies have been done to investigate the mechanism of neuronal infection by JCV. Here, we isolated primary cortical neurons from rat embryos and generated JCV growth curves by infecting neurons at MOIs of 0.1, 0.01, and 0.001. Supernatants were analyzed for viral RNA (RT-qPCR) and infectious titers (VPA). Primary neuron cultures were highly permissive to JCV in a dose-dependent manner, generating 10^5^–10^6^ PFU/mL of vRNA and infectious particles over time (Fig. 3A and B). These peak titers were similar to a previous study examining JCV replication in human NSCs and SH-SY5Y cells (36). Mock-infected cultures appeared healthy, containing neurons with long cellular processes staining prominently with βIII-tubulin (Fig. 3C). At an MOI of 0.1, JCV antigen staining was widespread by 24 hpi and remained prevalent at 48 hpi. While we did not directly examine cell death, as the infection progressed, the cellular debris in culture increased, resulting in a punctate βIII-tubulin staining pattern, indicating loss of neuronal structure by 60 hpi (Fig. 3C; Fig. S2A). Under the culture conditions used here, neurons expressed Lrp1 throughout the culture period (4 to 7 days in culture) (Fig. 3D; Fig. S3). Lrp1 expression was widely detectable by microscopy and was found in both the processes and cell bodies.

### Treatment of primary neurons with a high-affinity Lrp1 binding protein reduces JCV infection

**Fig 4:**
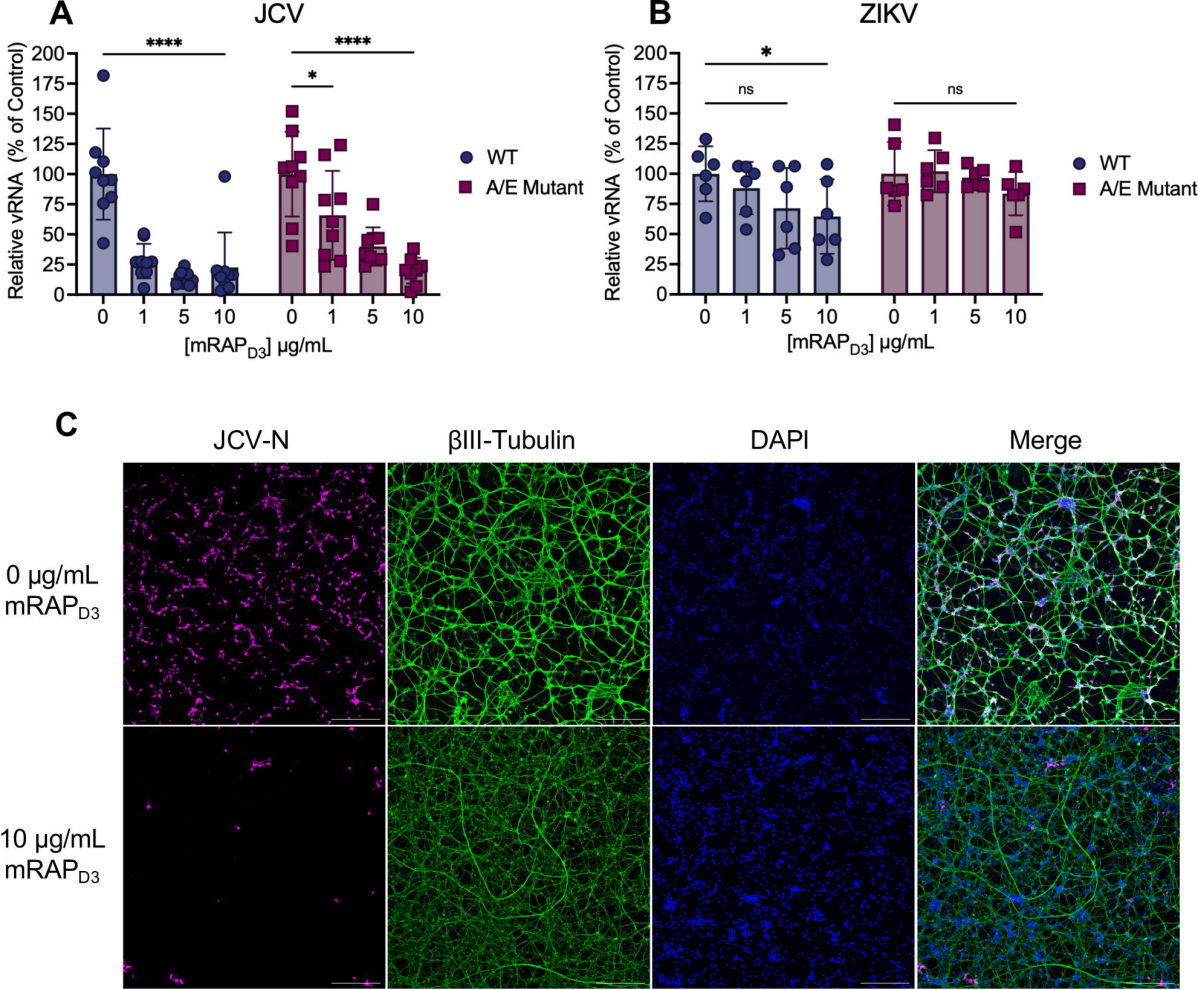
Pre-treatment with a high-affinity Lrp1 binding protein reduces JCV infection. Primary rat neurons were pre-treated with different concentrations of mRAP_D3_ for 45 min, followed by infection with JCV (**A**) or ZIKV MR766 (**B**) at an MOI of 0.1. Supernatant was collected for quantification of virus at 24 hpi (JCV) or 48 hpi (ZIKV). (**C**) Coverslips were stained for JCV-N (pink) and βIII-tubulin (green) and counterstained with DAPI (blue). Slides were imaged at 10× using a Leica DMI8 inverted microscope. Scale bar = 250 µm. Statistics were determined by two-way ANOVA with Dunnett’s multiple comparisons test. *, *P* < 0.05; ****, *P* < 0.0001; ns, no significance.

RAP is an intracellular high-affinity LRP1 chaperone protein known to competitively inhibit ligand binding to the CL_II_ and CL_IV_ domains of LRP1 (24). Domain 3 of mouse RAP protein (mRAP_D3_) can be added exogenously to cells prior to infection to interrogate reliance on LRP1 for infection, as we previously demonstrated with RVFV and OROV (15, 18). Here, primary neurons were pre-treated with recombinant mRAP_D3_ or a mutated version of mRAP_D3_ containing K265A/K279E mutations (A/E mutant), which reduces affinity for Lrp1 (15, 37), followed by infection with JCV (MOI = 0.1). At 24 hpi, viral RNA levels in the supernatant were reduced approximately 75%–90% in a dose-dependent manner compared to the infected untreated controls (Fig. 4A). The mutant mRAP_D3_, in comparison, was not as effective at reducing JCV viral RNA and decreased RNA titers only at the highest dose tested (10 µg/mL) (Fig. 4A). Plaque assays measuring infectious titer at 24 hpi showed a similar reduction to viral RNA after mRAP_D3_ treatment (Fig. S4A). By microscopy, viral antigen in mRAP_D3_-treated cells was restricted to small foci as opposed to being widespread throughout the culture in the untreated control images (Fig. 4C; Fig. S4B). When the assay was repeated with ZIKV, we found only a mild reduction in viral production at the highest dose with the WT mRAP_D3_, and no reduction in infection regardless of dose with the A/E mutant (Fig. 4B).

### Exogenous Gn protein from RVFV restricts JCV infection of primary neurons

**Fig 5:**
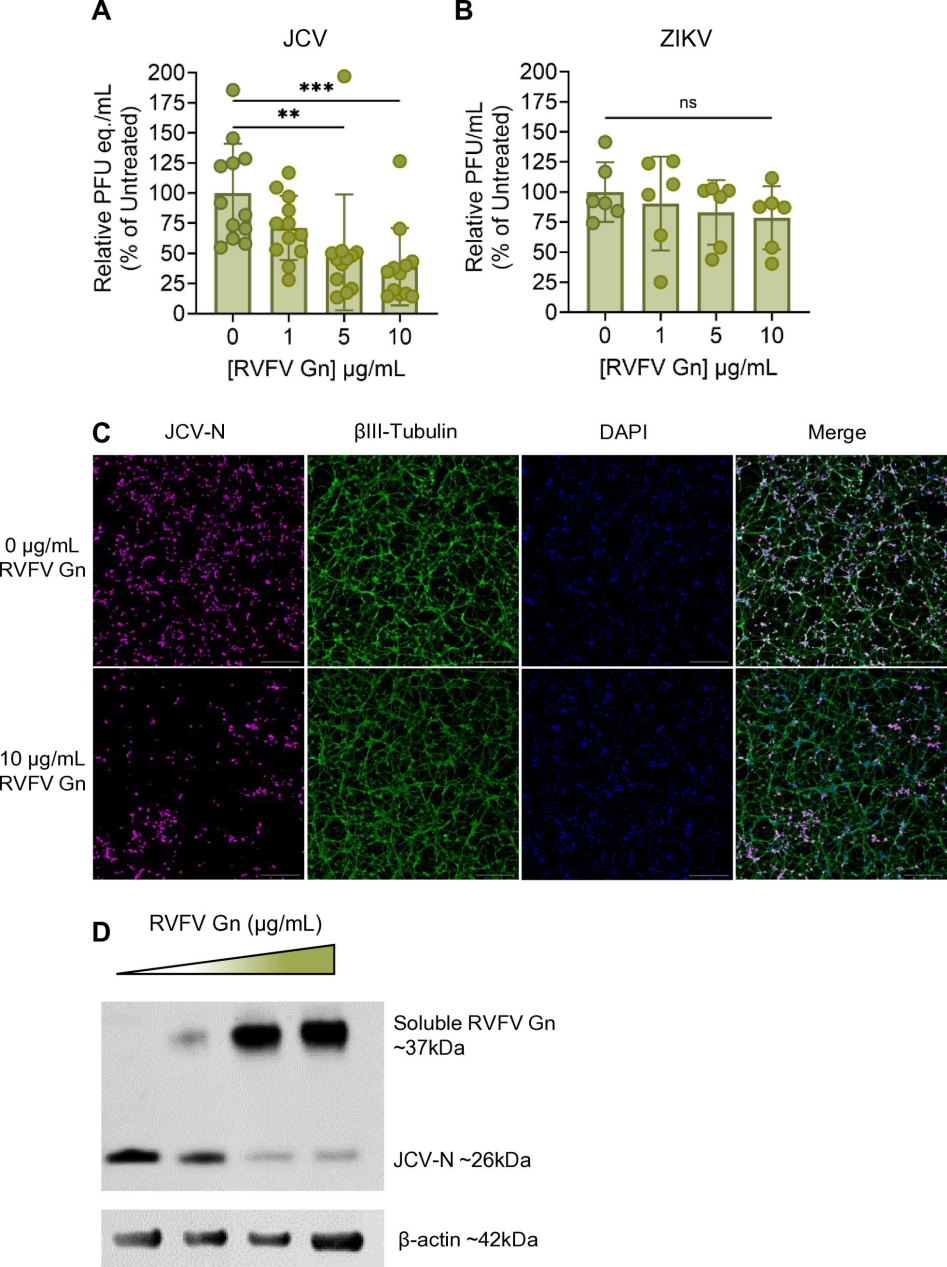
Pre-treatment with RVFV Gn reduces JCV infection. Primary rat neurons were pre-treated with different concentrations of recombinant, soluble RVFV Gn for 45 min, followed by infection with (**A**) JCV or (**B**) ZIKV MR766 at an MOI of 0.1. At 24 hpi, supernatant was collected for quantification of viral RNA. (**C**) Coverslips were stained for JCV-N (pink) and βIII-tubulin (green) and counterstained with DAPI (blue). Slides were imaged at 10× using a Leica DMI8 inverted microscope. Scale bar = 250 µm. (**D**) Cells were lysed in RIPA buffer and, to assess protein levels via Western blot, probed for RVFV Gn, JCV-N, and β-actin. Statistics were determined by one-way ANOVA with Dunnett’s multiple comparisons test. **, *P* < 0.01; ***, *P* < 0.001; ns, no significance.

The Gn glycoprotein of the distantly related bunyavirus RVFV binds to CL_II_ and CL_IV_ of LRP1, and exogenous treatment of cells with recombinant RVFV Gn competitively inhibited both homologous infection with RVFV and heterologous infection by OROV (15, 18). In a heterologous competition experiment to further probe the role of Lrp1 in JCV infection, primary neurons were pre-treated with recombinant RVFV Gn followed by infection with JCV. At 24 hpi, JCV titers were significantly reduced in the presence of 5 and 10 µg/mL of exogenous RVFV Gn (Fig. 5A). By Western blot, the amount of JCV-N protein detected in culture lysates decreased as increasing levels of RVFV Gn were added (Fig. 5D). Immunofluorescence microscopy revealed a decrease in viral antigen staining in cells treated with RVFV Gn compared to untreated cells (Fig. 5C; Fig. S2B). We observed no reduction in infection in the ZIKV controls (Fig. 5B). Our results indicating that RVFV Gn can competitively inhibit and reduce JCV infection suggest that JCV likely binds sites on Lrp1 CL_II_ and CL_IV_ that overlap with sites of RVFV Gn binding.

### Specificity of LRP1 for JCV entry

**Fig 6:**
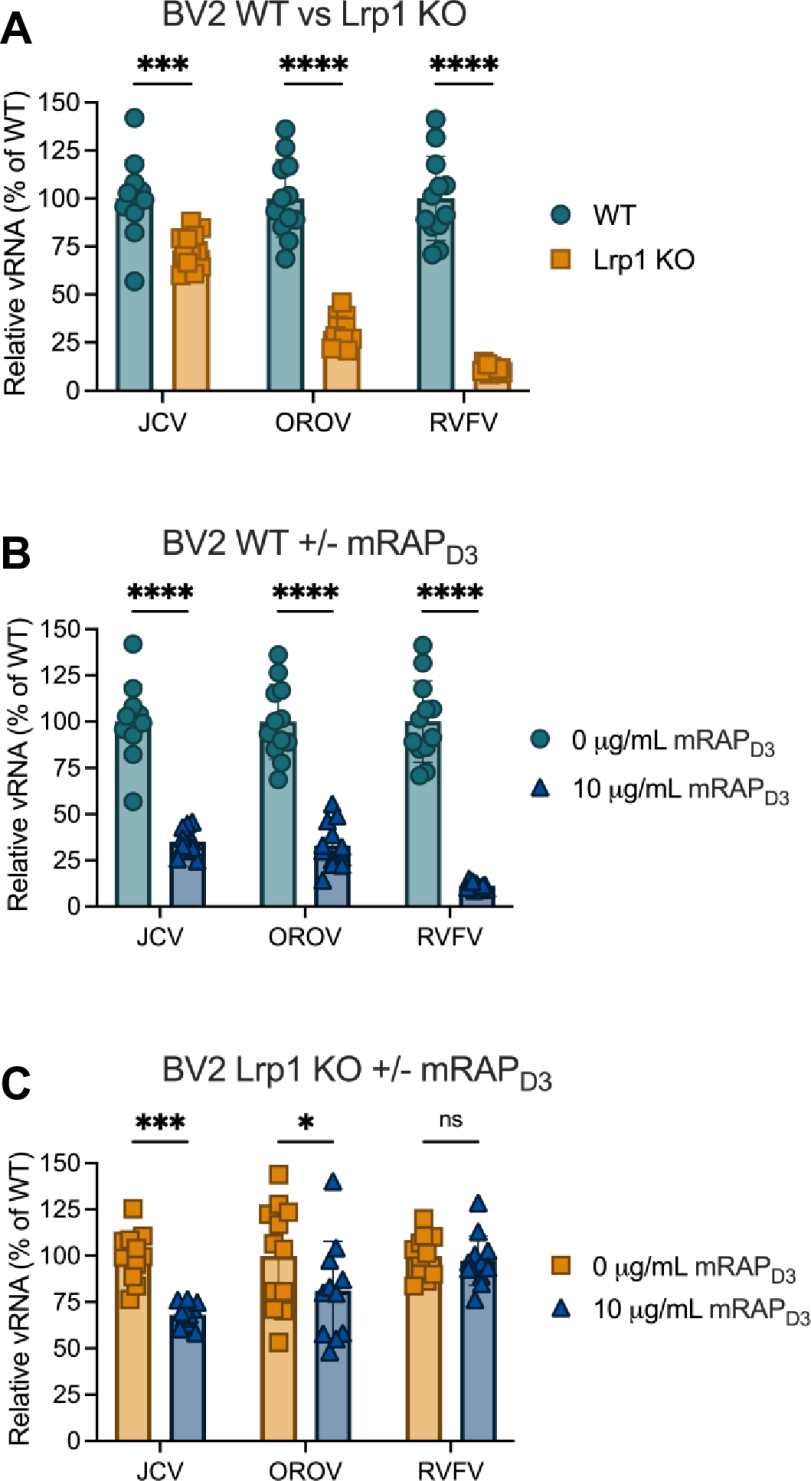
Specificity of Lrp1 for JCV entry. (**A**) BV2 WT and LRP1 KO cells infected with either JCV, OROV, or RVFV. (**B**) BV2 WT cells pre-treated with 0 or 10 µg/mL of mRAP_D3_, followed by infection. (**C**) BV2 Lrp1 KO cells pre-treated with 0 or 10 µg/mL of mRAP_D3_, followed by infection. Statistics were determined by two-way ANOVA with Dunnett’s multiple comparisons test. *, *P* < 0.05; ***, *P* < 0.001; ****, *P* < 0.0001; ns, no significance.

As we were unable to completely prevent infection by blocking or removing Lrp1, we investigated the role of other LDLR receptors in JCV infection. Multiple neurotropic alphaviruses can use more than one LDLR as a receptor (38–40), so we hypothesized that JCV may use another member of the LDLR family in addition to LRP1. One way to interrogate this is to treat Lrp1 KO cells with the high-affinity chaperone mRAP_D3_, which can bind several LDLRs (41). We pre-treated wild-type and Lrp1 knockout BV2 cells with 10 µg/mL of mRAP_D3_. When Lrp1 KO BV2 cells were infected with JCV, OROV, or the virulent RVFV strain ZH501, we observed a reduction in infection when compared to the WT cells, confirming our previous findings (15, 18) (Fig. 6A). When we pre-treated the WT cells with mRAP_D3_ followed by infection with each of these viruses, we again observed the expected decrease in titers across all three viruses (Fig. 6B). However, when the Lrp1 KO cells were pre-treated with mRAP_D3_, we observed a significant decrease in JCV infection, whereas much less or no decrease was seen with OROV or RVFV, respectively (Fig. 6C). These findings suggest that another LDLR may be involved in JCV infection in the absence of Lrp1, while infection by RVFV and OROV is likely primarily mediated by Lrp1.

## DISCUSSION

JCV is an arbovirus found in white-tailed deer and mosquitoes in North America. While severe disease in people is rare compared to overall seropositivity rates, the potential for further spread given deer-human proximity and the capacity to induce severe neurological clinical outcomes makes JCV an arbovirus of concern for the USA and Canada (4–7). At present, there is a significant knowledge gap in our understanding of the host factors that modulate infection of neurons. Immunocompetent mice have been used to study JCV neuropathogenesis; however, lack of neuroinvasion makes studying virus-cell interactions in the brain challenging (42). Intranasal and intracranial inoculation of JCV results in consistent neurologic disease in mice (36, 43), but this does not mimic a natural infection route, as JCV is primarily spread by mosquitoes. Mice deficient in type I interferon receptors or key signaling molecules (IRF3, IRF7, or MAVS) develop neurologic disease following intraperitoneal infection, suggesting that innate immunity is likely responsible for controlling JCV in the periphery and preventing neuroinvasion (44). In addition to our lack of understanding of antiviral host factors that restrict JCV *in vivo*, we also lack clarity regarding pro-viral cellular factors that mediate JCV infection at a cellular level.

The LDLR family of cell surface receptors is an evolutionarily conserved family of proteins with a variety of functions, including lipoprotein metabolism and cellular signaling (45). LDLRs have been implicated in mediating cellular entry of a variety of arboviruses, including multiple alphaviruses and bunyaviruses (15, 17–22, 38–40, 46). Many of these viruses have a wide host range and tissue tropism, which is supported by the evolutionary conservation and broad tissue distribution of the LDLR family members. LRP1 differs from other LDLRs that serve as viral receptors, such as LDLR, VLDLR, and ApoER2, in that it contains four ligand-binding cluster domains, while the other smaller family members are comprised of just one (47). This enables LRP1 to interact with ligands through multiple clusters, differentiating its interactions with ligands from the smaller members of the LDLR family (48). RVFV and OROV infections are supported by binding to CL_II_ and CL_IV_ (15, 18), and it is possible that both clusters interact with the multimeric viral glycoproteins during the course of attachment and entry through the cell membrane. But the specific steps in this process or the mechanism are incompletely described at present. The multimeric viral glycoprotein and the multitude of host factors involved in viral entry complicate our ability to define the exact molecular interactions between viruses and LRP1. Recently, SFTSV was shown to use LRP1 as an entry receptor in addition to the previously defined receptors CCR2 and NMMHC-IIA (17), highlighting the fact that arboviruses likely use multiple receptors or mechanisms to access cells. Interestingly, SFTSV binds to CL_I_ and CL_II_ of LRP1, further distinguishing this interaction on LRP1 from that of RVFV or OROV, which primarily use LRP1 CL_II_ and CL_IV_.

Neurons and other cells of the CNS express LRP1 (27), and LRP1 has a variety of critical functions in the brain, including the modulation of NMDA receptor signaling (49), neuronal glucose metabolism (50), and AMPA receptor stability (51). LRP1 has also been implicated in multiple neurodegenerative diseases, including Alzheimer’s disease, Parkinson’s disease, and Lewy body dementia (28–31). Other LDLRs also play important and often overlapping roles in the CNS. VLDLR and ApoER2 have been found to modulate synaptic plasticity (52) and neuronal migration (53). Mice with LRP1 deleted on a majority of their neurons (Lrp1^f/f^ Synapsin-Cre) display deficits in motor function (54), and VLDLR and ApoER2 double knockout mice display progressive hind limb paralysis and smaller brain size when compared to WT mice (53), demonstrating the importance of LDLR family members in the CNS function.

Given the conserved use of LRP1 by distantly related bunyaviruses RVFV, SFTSV, and OROV, we interrogated the dependence on LRP1 for infection by JCV. Our data shown here provide evidence that LRP1 is needed for efficient early-stage cellular infection by JCV. Furthermore, given the expression level and functionality of LRP1 in neurons, we determined its role in mediating JCV infection in primary neurons using an *ex vivo* primary rat neuron model in combination with the previously described molecular and biochemical tools (15, 18). Pre-treatment of primary neurons with two competitive inhibitors (mRAP_D3_ or heterologous recombinant Gn from RVFV) reduced JCV infection of primary rat neurons. The fact that RVFV Gn can inhibit JCV infection implies that these viruses may use overlapping regions on LRP1. Finally, we revealed evidence that JCV may use another unidentified member of the LDLR family, as mRAP_D3_ treatment reduced JCV infection in cells lacking Lrp1. Notably, we did not observe any further reduction in infection when this assay was repeated with RVFV, suggesting that Lrp1 is likely the primary member of the LDLR family involved in RVFV entry. Understanding which other LDLR family members can act as a receptor for JCV will be the focus of future studies.

Future studies will focus on the molecular mechanisms by which JCV engages with LRP1. While RVFV binds to LRP1 through interactions with the surface glycoprotein Gn (15), there are large differences in the glycoprotein structures of viruses within *Bunyavirales* (55). Additionally, Crimean-Congo hemorrhagic fever virus, a more distantly related bunyavirus in *Nairoviridae*, interacts with LDLR through the Gc glycoprotein (20, 21). Therefore, JCV may engage LRP1 in a different manner than RVFV does, including potential binding by Gc rather than Gn. Additional studies are warranted to further clarify the mechanism of JCV-LRP1 binding.

In summary, we present evidence that LRP1 is a host factor involved in the early stages of cellular infection by JCV. The combination of this work with previously published results by our groups and others on RVFV, OROV, and SFTSV underscores the importance of LRP1 as a multi-bunyaviral host factor. The fact that LRP1 is highly conserved and is utilized in early infection of diverse bunyaviruses makes it an attractive target for the development of broad bunyavirus therapeutics.

## Data Availability

All data needed to evaluate the conclusions in the paper are included in the main paper and supplemental material. Reagents are available upon request from the corresponding authors.

## SUPPLEMENTAL MATERIAL

Supplemental figures jvi.01841-25-s0001.pdf Figures S1 to S4.

ASM does not own the copyrights to Supplemental Material that may be linked to, or accessed through, an article. The authors have granted ASM a non-exclusive, world-wide license to publish the Supplemental Material files. Please contact the corresponding author directly for reuse.
