# Supplementary material for: LRP1 facilitates Jamestown Canyon virus infection of neurons
Source: J Virol. 2025 Nov 28;99(12):e01841-25. doi: 10.1128/jvi.01841-25 (PMC12724265; doi:10.1128/jvi.01841-25)
Supplement: Supplemental figures — Figures S1 to S4. [file jvi.01841-25-s0001.pdf]

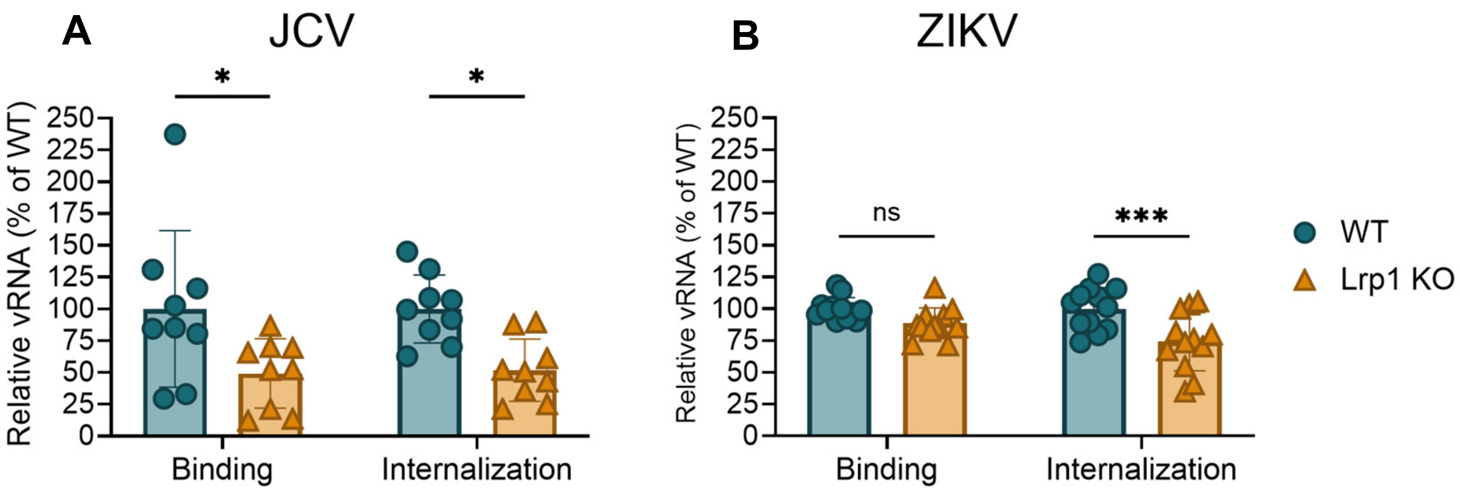

Supplemental Figure 1. Vehicle controls for binding and internalization experiments. WT and Lrp1 knockout BV2 cells were incubated with DMSO diluted in PBS at 4°C for 30 minutes in the absence of surfen. The solution was removed and cells were incubated with JCV (A) or ZIKV (B) at an MOI of 0.1 for 1 hour at 4°C. Cells were washed and binding samples were collected. Cells were returned to 37°C for 1 hour and internalization samples were collected. Statistics determined by two-way ANOVA.

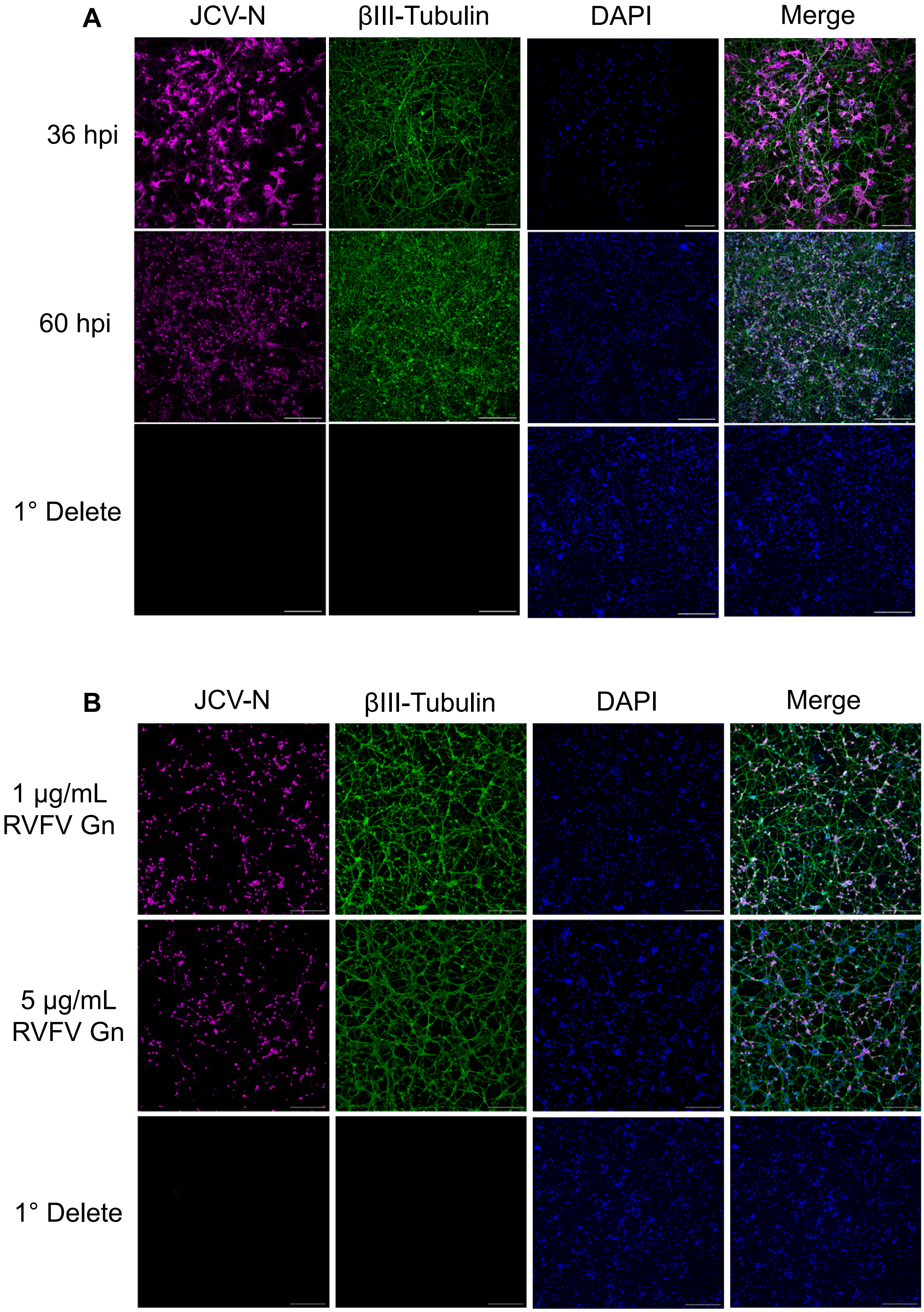

Supplemental Figure 2. Additional images of immunofluorescent microscopy from Figures 3 and 5. (A) Additional images of 36 hpi, 60 hpi, and primary delete of JCV infected primary rat neurons. Slides were imaged at 20X using a Nikon A-1 confocal microscope. Scale bar = 250µm. (B) Additional images of RVFV Gn treatment of primary rat neurons, including primary delete. Slides were imaged at 10X using a Leica DMI8 inverted microscope. Coverslips were stained for JCV-N (pink) and βIII-Tubulin (green) and counterstained with Hoescht (blue). Scale bar = 250µm.

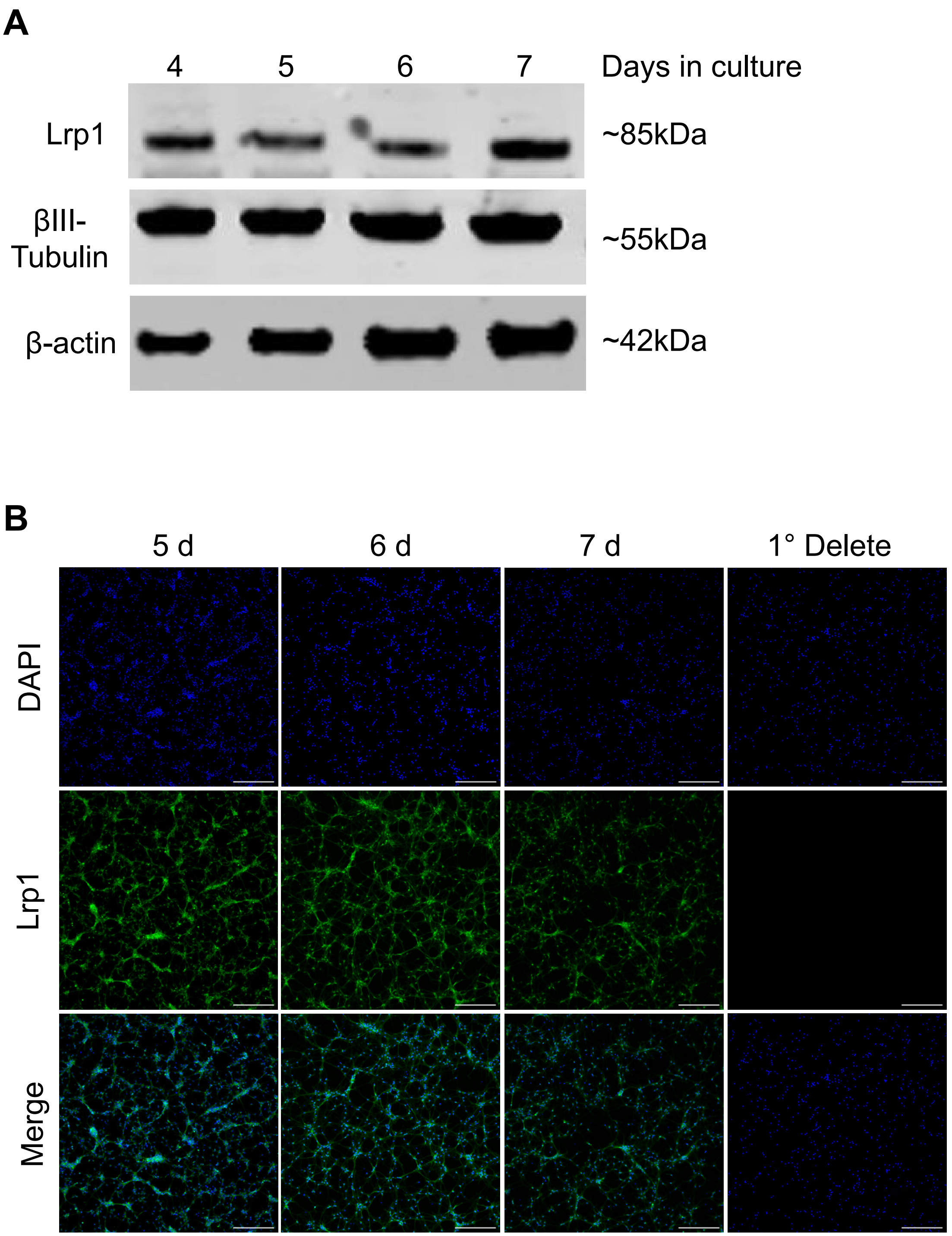

Supplemental Figure 3. Lrp1 expression shown by Western blot and additional images of immunofluorescent microscopy from Figure 3. (A) Western blots probing for the indicated proteins in lysates from primary neurons at 4-7 days in culture. (B) Additional images showing Lrp1 expression in primary rat neurons across days 5, 6, and 7 in culture. Coverslips were stained for Lrp1 (green) and counterstained with DAPI (blue). Slides were imaged at 10X using a Leica DMI8 inverted microscope. Scale bar = 250μm.

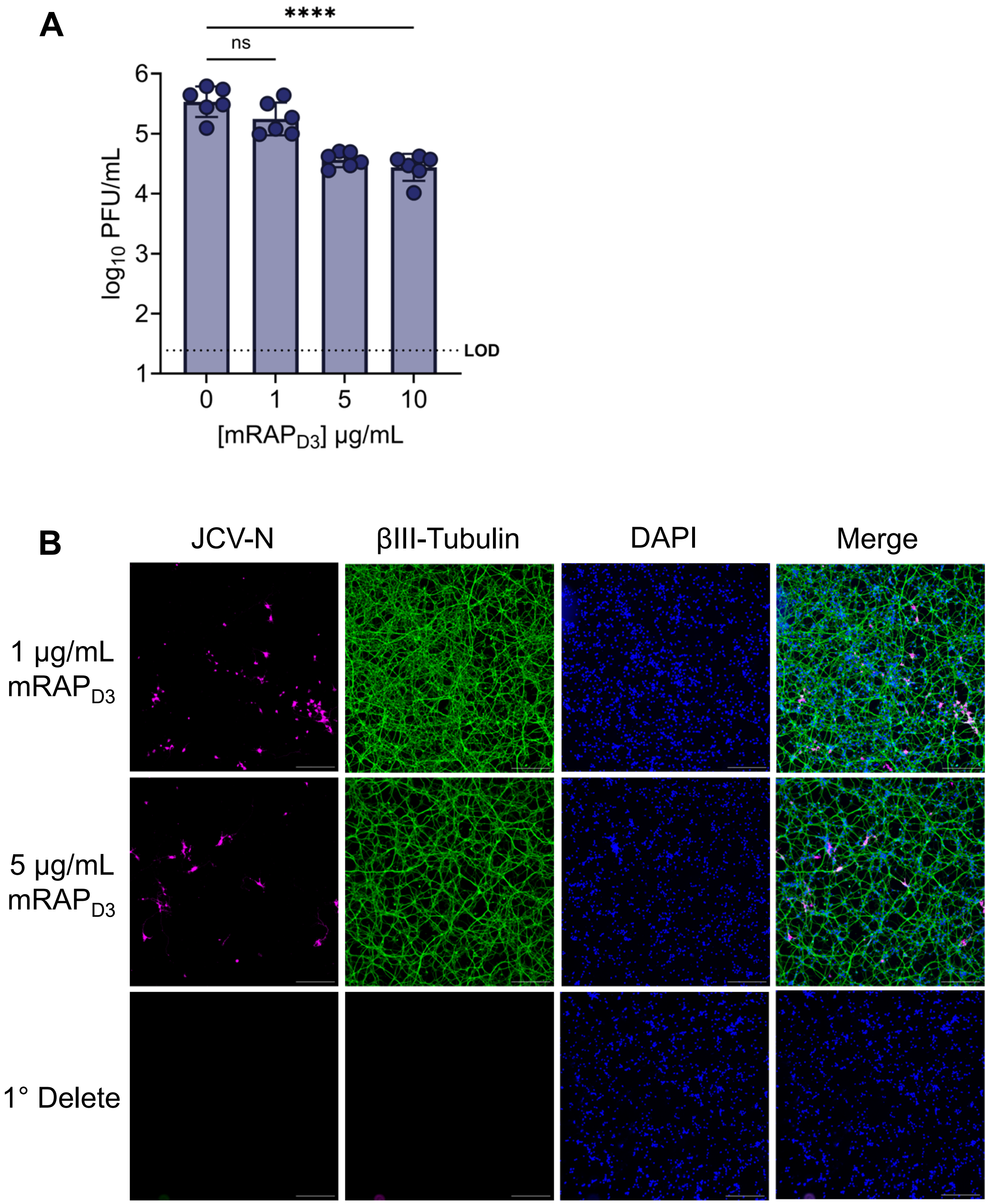

Supplemental Figure 4. $mRAP_{D3}$ treatment and additional images of immunofluorescent microscopy from Figure 4. (A) Infectious titers from primary rat neurons treated with WT $mRAP_{D3}$. (B) Additional images of $mRAP_{D3}$ treatment of primary rat neurons, including primary delete. Coverslips were stained for JCV-N (pink) and βIII-Tubulin (green) and counterstained with Hoescht (blue). Slides were imaged at 10X using a Leica DMI8 inverted microscope. Scale bar = 250µm. Statistics determined by one-way ANOVA with Dunnett's multiple comparison test.
